# The Diagnostic Accuracy of LOGIQ S8 and E9 Shear Wave Elastography for Staging Hepatic Fibrosis, in Comparison with Transient Elastography

**DOI:** 10.3390/diagnostics11101817

**Published:** 2021-10-01

**Authors:** Jeong-Ju Yoo, Sang Gyune Kim, Young Seok Kim

**Affiliations:** Division of Gastroenterology and Hepatology, Department of Internal Medicine, Soonchunhyang University, Bucheon Hospital, Bucheon 14584, Korea; puby17@naver.com (J.-J.Y.); liverkys@schmc.ac.kr (Y.S.K.)

**Keywords:** chronic liver disease, ultrasonography, shear wave elastography, liver stiffness

## Abstract

Background: The aim of this study was to evaluate the usefulness of two different types of 2-dimensional shear wave elastography (2D-SWE) for predicting liver fibrosis stages in comparison to transient elastography (TE), using a histologic METAVIR scoring system as the reference method. Methods: A total of 203 patients with chronic liver disease were prospectively enrolled in the study. Two different 2D-SWEs (LOGIQ S8 and E9 systems, GE Healthcare, Chalfont St Giles, UK) were assessed for liver stiffness in patients with chronic liver diseases. Patients received 2D-SWE examinations with the S8 and E9 systems, and also underwent TE (FibroScan^®^, Echosens, France) tests and liver biopsies on the same day. Results: The most common etiology of chronic liver disease was non-alcoholic fatty liver disease (28.7%), followed by chronic hepatitis B (25.1%). Liver fibrosis stages consisted of F0 (22.6%), F1 (29.7%), F2 (16.9%), F3 (12.8%) and F4 (17.9%). Overall, S8 and E9 were well correlated with the histologic fibrosis stages. The optimal cut-off values for S8 and E9 to differentiate significant fibrosis (≥F2) were 6.70 kPa and 6.42 kPa, respectively, while the cut-off values for S8 and E9 in distinguishing liver cirrhosis were 9.15 kPa and 8.88 kPa, respectively. Among the 195 patients who had successful measurements in both S8 and E9, liver stiffness showed good inter-equipment correlation (ICC: 0.900, *p* < 0.001). Regarding diagnostic ability, upon comparison (FibroScan^®^), there were no significant differences between 2D-SWEs and TE for detecting every stage of liver fibrosis. Conclusion: In comparison to TE, 2D-SWE with LOGIQ S8 and E9 (GE Healthcare) are useful non-invasive tools for predicting significant fibrosis and liver cirrhosis.

## 1. Introduction

Chronic liver diseases (CLDs) are a major global health issue that require increased awareness and effort for effective management and treatment [1]. Liver fibrosis, the product of persistent liver damage, is the most important prognostic factor in patients with CLDs [2]. Increases in the prevalence of CLD have given rise to the importance of non-invasive tools for liver fibrosis estimation [3]. Although liver biopsy is the gold standard for identifying the stage of fibrosis, it is an invasive method with poor patient acceptance. Overall, major adverse events associated with liver biopsy are reported to be rare (0.05%) [4], but it can lead to fatal complications such as major bleeding or even death [4,5,6]. In addition, the histological samples obtained may be incomplete, and discrepancies may occur among the examiners [7,8].

Therefore, many studies have been conducted to measure liver fibrosis with less invasive methods, which are largely divided into serological tests and non-serological tests. Serological tests include Fibrosis-4 index [9], NAFLD fibrosis score [10], AST to Platelet Ratio Index (APRI) score [11], and enhanced liver fibrosis [12,13], which combine various factors reflecting liver fibrosis. Non-serological tests include transient elastography (TE) (FibroScan^®^, Echosens, France) [14,15] and magnetic resonance elastography (MRE) [16].

Among such minimally invasive tests, TE has been most widely used in recent years due to its non-invasive method, reproducibility, and ability to numerically quantify the degree of liver fibrosis [17]. In addition, TE can predict not only the diagnosis of fibrosis, but also the occurrence and prognosis of various complications of cirrhosis. [18,19] However, liver stiffness measurement by TE requires additional expensive equipment other than the existing ultrasonic tool. In addition, it is difficult for the operator to directly identify the region of interest during the examination process, and thus the success rate decreases in patients with abdominal obesity, mass or ascites [20]. During the last 10 years, two-dimensional shear wave elastography (2D-SWE) technique had emerged as another noninvasive method to assess fibrosis, and is widely used in clinical practice [21,22,23,24]. 2D-SWE is easily integrated into the routine B-mode examination transducers and the examiner can check the grayscale B-mode images and SWE results simultaneously. LOGIQ S8 (GE Healthcare, Wauwatosa, WI, USA) is one of the recently developed tools for ultrasound examination that is capable of the 2D-SWE technique.

2D-SWE applies a perpendicular stress force on the liver to induce shear on the tissue, and the machine measures the shear wave velocity in an ultrasonic system. This velocity varies depending on the degree of liver fibrosis, and through this, the degree of fibrosis can be predicted [21,22,23,24].

S8 and E9 2D-SWEs share the same principle of liver fibrosis measurement using ARFI technique as other 2D-SWEs [21,22,23,24]. However, unlike TE, S8 and E9 can directly set the region of interest while viewing the 2D image. The acoustic radiation force impulse occurs in four places within the probe, so that the measured value is more accurately measured over a wider range. As such, S8 and E9 2D-SWEs have many advantages compared to TE. However, studies on the reliability and validity of this equipment are few in number [25,26,27].

Therefore, the aim of this study was to evaluate the usefulness of two different models of 2D-SWE, S8 and E9 from GE Healthcare, for predicting liver fibrosis stages. In addition, a comparative analysis was conducted between 2D-SWE and TE (Fibroscan^®^) using the histologic METAVIR scoring system as the reference method.

## 2. Materials and Methods

### 2.1. Patients and Study Endpoint

Between October 2017 and June 2019, a total of 203 patients with chronic liver disease were prospectively enrolled in a referral tertiary care hospital. The subjects of this study were patients with chronic liver disease, and their liver stiffness was measured by three methods: S8 2D-SWE, E9 2D-SWE and transient elastography Fibroscan^®^. The inclusion criteria for the subjects were as follows; (1) aged 19–70 years, (2) underwent 10 continuous measurements of SWE, (3) underwent TE test and liver biopsy within a month of SWE, (4) possible to calculate AST to Platelet Ratio Index (APRI), (5) agreed to measure liver stiffness using LOGIQ S8. The exclusion criteria were as follows; (1) liver cirrhosis with Child–Pugh class B, C, (2) complications such as ascites, spontaneous bacterial peritonitis, hepatic encephalopathy or variceal bleeding, (3) diagnosed with hepatocellular carcinoma or cholangiocarcinoma within the last 5 years, (4) possibility of congestive hepatopathy due to kidney failure, end-stage renal disease, or congestive heart failure, (5) disease of gallbladder or biliary tract disease, (6) impossible to measure liver stiffness due to dermatological problems, or (7) AST or ALT > 200 IU/L, or total bilirubin ≥ 5 mg/dL.

The primary endpoint of this study was the comparison of diagnostic accuracy for liver fibrosis stage based on liver stiffness measured by LOGIQ S8, E9 and TE. The study protocol was approved by the Institutional Review Board of Soonchunhyang University Bucheon Hospital (SCHBC 2017-08-002-001, date of registration 27 September 2017), and conformed to the ethical guidelines of the World Medical Association Declaration of Helsinki. Written informed consent was obtained from all patients participating in the study.

### 2.2. Liver Histology and Transient Elastography

Liver specimens were fixed in formalin and embedded in paraffin. Sections were stained with hematoxylin–eosin and Masson’s trichrome. Each biopsy specimen was analyzed by two experienced pathologists. Fibrosis was assessed to be at a stage from 0 to 4 on a scale using the following METAVIR criteria: F0, no fibrosis; F1, portal fibrosis without septa; F2, periportal fibrosis; F3, septal fibrosis; F4, liver cirrhosis [28].

Liver stiffness was measured by Fibroscan^®^ using M probe or XL probe according to the patient’s status. In our study, the XL probe was applied to 12 patients. The success rate of TE was calculated as the number of valid measurements divided by the total number of measurements. Ten measurements were performed with a success rate of at least 60%. The median value was taken as the representative value. The interquartile range (IQR) was defined as the index of intrinsic variability in the LS values corresponding to the interval between the 25th and 75th percentiles, which contains 50% of the valid LSMs taken. Only procedures with at least ten valid measurements and IQR/median value <0.3 were considered. TE was performed by expert physicians with experience conducting such tests on more than 1000 cases.

### 2.3. Shear Wave Elastography

Two different 2D-SWEs (LOGIQ S8 and E9 systems, GE Healthcare, Wauwatosa, WI, USA) were assessed for liver stiffness measurements in patients with CLDs. 2D-SWE examinations were performed during abdominal ultrasound. 2D-SWEs were performed by three hepatologists with more than 5 years of experience on more than a thousand examinations. Patients should fast for 8 h and rest for a minimum of 10 min before undergoing liver stiffness measurement. Liver stiffness measurement by SWE was performed through a right-side intercostal space in supine position, with the right arm in extension, with breath held and at least 10 mm below the liver capsule. Ten measurements were taken and an IQR/M ≤ 30 % of the 10 measurements is considered a reliable value [21].

### 2.4. Sample Size Calculation

This study attempted to compare sensitivity and specificity using ROC curves to determine the diagnostic accuracy of LOGIQ S8 and Fibroscan^®^ for predicting advanced liver fibrosis (≥F3). To our knowledge, there have been no prior studies on the diagnostic accuracy of LOGIQ S8 compared with LOGIQ E9 and TE. Therefore, we assumed that the AUC of Fibroscan^®^ was 0.83 and the AUC of LOGIQ S8 was 0.90. When the alpha error was set to 0.05 and the power was 0.8, and the unreliable measurement was estimated to be 10%, the total sample size was 195.

### 2.5. Statistical Analysis

Frequencies and percentages are used for the descriptive statistics. Significant differences between the groups were investigated using the χ^2^ test for categorical variables and Student’s t-test for continuous variables. Reliability was assessed using Lin’s concordance correlation coefficient. The diagnostic performance was calculated using the area under the receiver operating characteristics curve (AUROC). To confirm the association between the two tools, correlation analysis using Pearson’s correlation coefficient and intraclass correlation coefficient (ICC) was performed. Diagnostic performance was compared using DeLong’s method. All statistical analyses were performed using R version 4.3.1 (The R Foundation for Statistical Computing, Vienna, Austria). Statistical significance was set at *p* < 0.05.

## 3. Results

### 3.1. Baseline Characteristics

Among the total of 203 patients who underwent S8 and E9 2D-SWE, four patients experienced technical failure during measurement trials and four patients showed unreliable measurement results. Technical failure and unreliable measurement rates were 1.9% and 1.9%, respectively. The overall success rate of S8 was 96.1% (195/203). There was no significant difference in success rates between the 2D-SWE and TE system.

After excluding the aforementioned eight patients, a total of 195 patients were included in the analysis (Table 1). The mean age of the patients was 47.98 ± 13.98 years, and 109 (55.9%) patients were male. The most common etiology of chronic liver disease was non-alcoholic fatty liver disease (NAFLD) (28.7%) followed by chronic hepatitis B (25.1%). Liver fibrosis stages evaluated by histology consisted of F0 (22.6%), F1 (29.7%), F2 (16.9%), F3 (12.8%) and F4 (17.9%).

### 3.2. Diagnostic Performances of S8 2D-SWE, E9 2D-SWE and TE for Distinguishing Liver Fibrosis Stage

**Figure 1:**
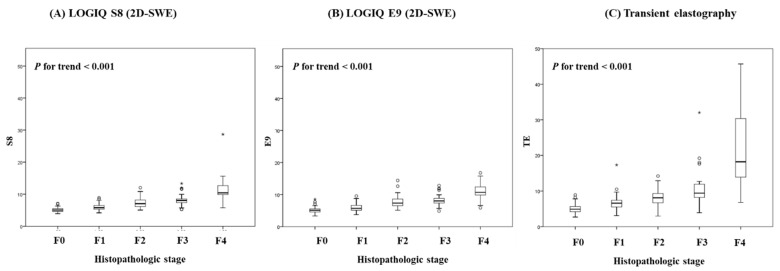
Box plots of 2D-SWEs for each Liver Fibrosis METAVIR Stage. (**A**) LOGIQ S8, (**B**) LOGIQ E9, (**C**) Transient elastography.

Overall, S8 2D-SWE, E9 2D-SWE and TE were well correlated with histologic fibrosis stages. As the histological grade increased, the values of S8 (Figure 1A), E9 (Figure 1B) and TE (Figure 1C) significantly increased (both *p* for trend <0.01). When examining the correlation coefficient between the histologic stage and LS measured by S8, E9 and TE, it was 0.841, 0.726 and 0.623, respectively, with all *p* levels < 0.001, and 0.819, 0.840 and 0.726 in the comparisons between S8 and E9, between S8 and TE and between E9 and TE, respectively (all *p* < 0.001) (Table 2).

Finally, we compared the diagnostic performances of both 2D-SWE systems compared with TE. As has been reported many times before, TE predicted F4 (AUROC 0.951, 95% CI 0.909–0.981) with the best capability, and virtually predicted all fibrosis stages well above AUROC 0.9 (Table 3). When comparing diagnostic ability with 2D-SWE and TE, no significant differences were shown in detecting each stage (all *p* > 0.05) (Supplementary Table S1).

**Figure 2:**
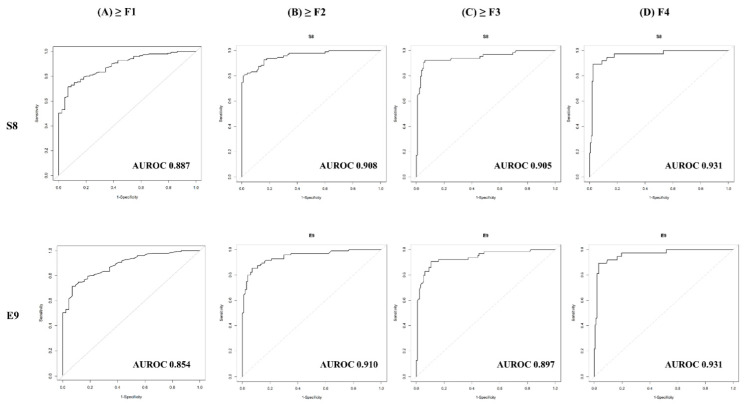
ROC curves for 2D-SWE for difference in fibrosis stages. (**A**) F0 versus F1-F4 (≥F1), (**B**) F0-F1 versus F2-F4(≥F2), (**C**) F0-F2 versus F3-F4(≥F3), (**D**) F0-F3 versus F4(F = 4).

We calculated the cut-off value of the two types of 2D-SWE (S8, E9) predicting ≥F1, ≥F2, ≥F3, and F4 stages. The optimal cut-off value of S8 and E9 to differentiate significant fibrosis (≥F2) were 6.70 kPa and 6.42 kPa, respectively (Table 3). The cut-off values of S8 and E9 for distinguishing advanced fibrosis (≥F3) were 7.25 kPa and 7.35 kPa, respectively. The cut-off values of S8 and E9 for distinguishing liver cirrhosis (F4) were 9.15 kPa and 8.87 kPa, respectively. These results illustrate that S8 and E9 have almost similar cut-off criteria for distinguishing liver fibrosis stages. In the prediction of any fibrosis stage in both S8 and E9, the AUROC was as high as 85%. The ROC curves for 2D-SWE for differentiating fibrosis stages ≥F1, ≥F2, ≥F3, and F4 are depicted in Figure 2. Different cut-off values according to etiology (viral vs. non-viral) are presented in Supplementary Table S1.

Based on the S8 and E9 2D-SWE cut-off value, we compared the fibrosis stage classified by histologic stage and by S8 and E9 liver stiffness scores (Table 4). Of the 195 patients, 105 patients (54%) in S8 and 105 patients (54%) in E9 showed findings consistent with the result of liver biopsy. In 90 patients (46%) of S8 and E9, the fibrosis stage classification did not match. Concordance using kappa value was 0.635 (95% CI 0.564–0.706) in S8 and 0.423 (95% CI 0.381–0.465) in E9. Similarly, in TE, fibrosis stage classification was consistent in 105 patients (54%), with kappa value 0.422 (95% CI 0.333–0.511, *p* < 0.001).

### 3.3. Interobserver Reliability between Two Types of 2D-SWEs, S8 and E9

The correlation coefficient between S8 and E9 was 0.819 (Table 2, *p* < 0.001). The ICC for inter-equipment agreement between S8 and E9 was 0.900 (95% CI 0.867–0.924, *p* < 0.001). The Bland–Altmann plot is presented in Supplementary Figure S1.

### 3.4. Diagnostic Performances in Comparison with Transient Elastography

**Figure 3:**
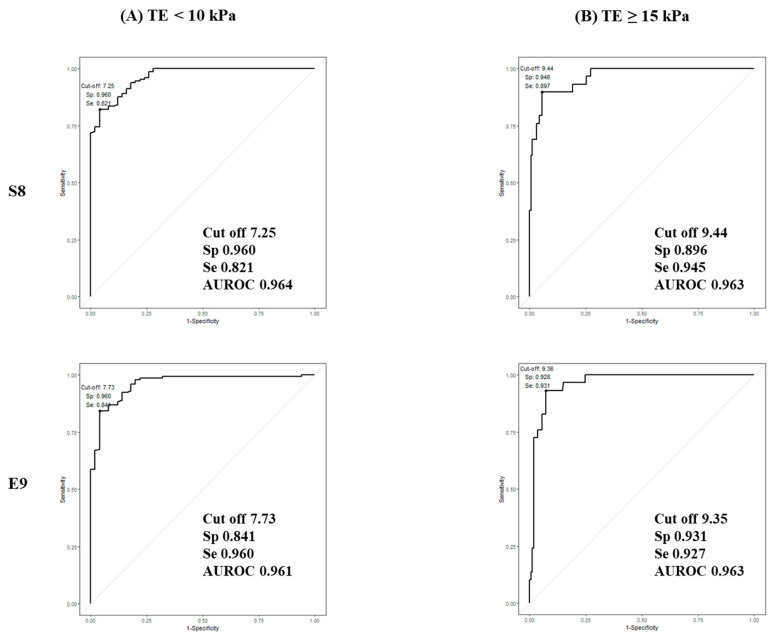
ROC curves for 2D-SWE for predicting Fibroscan^®^. (**A**) Fibroscan^®^ < 10 kPa, (**B**) Fibroscan^®^ ≥ 15 kPa.

In Baveno VI criteria, the cut-off value for TE predicting compensated advanced chronic liver disease (cACLD) is between 10 and 15 kPa [29]. Thus, we calculated the cut-off of the S8 and E9 2D-SWE to predict TE < 10 kPa and TE ≥ 15 kPa (Table 5). The cut-off value for S8 and E9 predicting TE <10 kPa was 7.25 kPa (AUC 96.4%), 7.73 kPa (AUC 96.1%), respectively. The cut-off value for S8 and E9 predicting TE ≥ 15 kPa was 9.44 kPa (AUC 96.3%), 9.35 kPa (AUC 96.3%), respectively. The ROC curves are shown in Figure 3.

### 3.5. Correlation among Values Obtained by APRI Index and Platelet Count and LS by S8, E9 and TE

**Figure 4:**
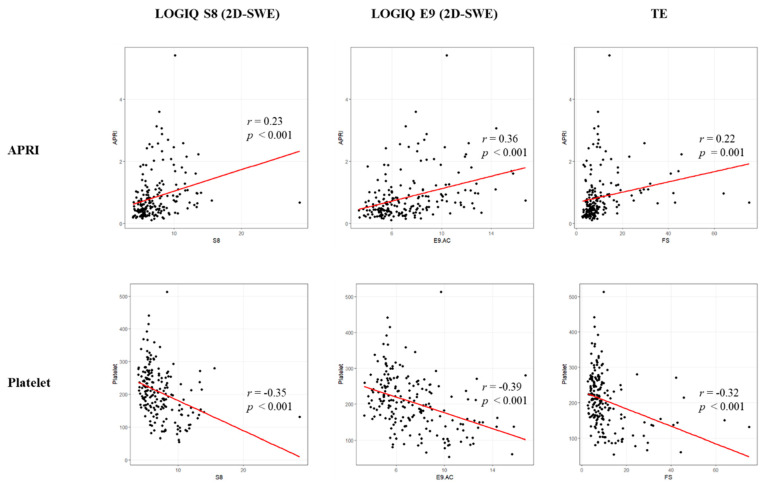
Correlation between APRI, platelet count and liver stiffness (S8, E9, and TE).

Lastly, we performed correlation analysis to compare the APRI index/platelet count and liver stiffness. The APRI indices were significantly positively correlated with the LS by S8 (r = 0.23, *p* < 0.001), E9 (r = 0.36, *p* < 0.001) and TE (r = 0.22, *p* = 0.001). On the other hand, platelet count and liver stiffness showed a significant negative correlation in S8 (r = −0.35, *p* < 0.001), E9 (r = −0.39, *p* < 0.001), and TE (r = −0.32, *p* < 0.001) (Figure 4).

## 4. Discussion

Recently, various 2D-SWEs have been developed by a number of companies. However, studies comparing TE and 2D-SWE head-to-head using liver histology are rarer [30,31]. Our study confirmed that the two models of 2D-SWE (GE LOGIQ S8, LOGIQ E9) not only accurately detect histological fibrosis in various liver diseases, but also have a high correlation with TE. This indicates that 2D-SWE can be used to measure liver stiffness by substituting for or supplementing TE for long-term follow-up or monitoring of patients with liver disease. However, few papers have prospectively compared various SWE tools, such as in our study.

Technical failure and unreliable measurement rates of 2D-SWE were 1.9%in this study, which was relatively low. In the meta-analysis, the technical failure of 2D-SWE was reported as 2.2%, which was significantly lower than that of TE (12.1%) or 1D-SWE (4.1%) [32]. This is probably due to the characteristics of 2D-SWE that can be directly measured while observing B-mode images. 2D-SWE can also be conducted, to some extent, on patients with ascites and obesity, which is not possible with TE.

There have been several studies on the relationship between 2D-SWE and liver histology that have shown a high degree of agreement regardless of etiology [33,34]. In most studies, the AUROC of 2D-SWE performance was higher than 0.9 in the prediction of fibrosis stage. A meta-analysis based on individual patients data showed that the AUROCs of 2D-SWE in patients with hepatitis C, hepatitis B, and nonalcoholic fatty liver disease were 86.3%, 90.6%, and 85.5% for diagnosing significant fibrosis, and 92.9%, 95.5%, and 91.7% for diagnosing cirrhosis, respectively [31]. Existing studies regarding the relationship between 2D-SWE and liver histology included various manufacturers and models, but did not evaluate the model (LOGIQ S8 and E9) used in this study.

However, the correlation between 2D-SWE and TE shows a high degree of agreement in most of the studies [35,36,37,38,39]. In addition, the correlation between the recently developed MR elastography and 2D-SWE is reported to be high, so it is expected to be useful in clinical practice [34,40,41].

The cut-off for 2D-SWE used to estimate liver fibrosis stage is reported differently in each study, probably due to the wide distribution of etiologies of liver disease. In our study, chronic hepatitis B and NAFLD account for the majority, and cut-offs of F2, F3, and F4 of S8 2D-SWE were 6.70 kPa, 7.26 kPa, 9.15 kPa, respectively. In a recently published patient data-based meta-analysis, the proposed cut-offs for ≥F2, ≥F3 and F4 of 2D-SWE were 7.1 kPa, 8.1 kPa, and 11.5 kPa in chronic hepatitis B, and 7.1 kPa, 9.2 kPa, and 13.0 kPa in NAFLD, respectively [31]. In our study, the cut-off predicting ≥F2 and ≥F3 is similar, but the cut-off of F4 tends to be lower. This is due to the fact that body mass index in the patients in our study was relatively low (mean BMI 25.1) and 81% of patients with F4 had normal ALT levels. In the S8 study also using GE LOGIQ S8, cut-offs of ≥F2, ≥F3, and F4 were reported as 6.9 kPa, 8.2 kPa, and 9.3 kPa, which is similar to our study [25].

It has yet to be concluded whether a different cut-off value should be applied depending on the company or model, even with identical 2D-SWE principles. In one study, it was reported that the S8 and E9 manufactured by the same company had almost the same cut-off, so interchangeability was possible even if the models were different [25]. However, other studies concluded that the cut-off was different for each model, so that interchangeability was not possible [27]. There is a need for future research on validation with models of various 2D-SWE manufacturers.

Lastly, our study confirmed that liver stiffness measured by different methods correlated significantly well with APRI (positive correlation) or platelet count (negative correlation). APRI score and platelet count have proven to be useful in predicting not only liver fibrosis, but also clinical prognosis and complications of portal hypertension. Thus, 2D-SWE and TE are regarded as useful not only to obviate the need for liver biopsy in high-risk populations, but also to predict the prognosis in these patients. In fact, many studies have shown that TE is helpful in predicting long-term prognosis [42,43], and we believe that studies related to SWE and long-term prognosis should be conducted in the future.

The advantage of this study was that the cut-off of 2D-SWE for each model was suggested based on liver histology. In addition, all liver stiffness measurements using the two types of 2D-SWE and TE were performed on the same day. Interchangeability is particularly important in patients at high risk for hepatocellular carcinoma (HCC) and those who continue to require surveillance for HCC. For such patients, ultrasound as a surveillance test is recommended every 6 months. If SWE is available, additional information on liver fibrosis can be obtained by performing ultrasound surveillance without the need to purchase a separate expensive machine.

There were several limitations to our study. First, not every cut-off according to etiology could be stated, as a range of liver diseases were included in this study. Second, the standard method for liver stiffness measurement by 2D-SWE has yet to be firmly established, and thus other studies may report different reproducibility results. In order to minimize such shortcomings in this study, we adopted a suitable measurement after all the researchers performing 2D-SWE cross-validated each other for correct measurements. Third, our study did not analyze the effects of different liver pathologies causing liver fibrosis on the measurement of SWE. Finally, we could not observe the prospective clinical outcomes of patients according to the value of liver stiffness. Although it is uncertain how useful 2D-SWE would be to predict the prognosis, TE accurately estimated compensated advanced chronic liver disease.

## 5. Conclusions

In conclusion, S8 and E9 2D-SWEs have a high correlation with liver histology and TE, so they can be clinically used to predict fibrosis stages in various liver diseases. It is expected that the accumulated data of 2D-SWE based on liver biopsy will provide more standardized reference values for predicting fibrosis stages in the future.

## Figures and Tables

**Table 1:** Baseline characteristics of the patients.

Variables	*n* = 195
Age (years)	
Mean ± SD	47.98 ± 13.98
Gender	
Male Female	109 (55.9%) 86 (44.1%)
Etiology	
HBV	49 (25.1%)
Alcoholic liver disease	34 (17.4%)
NAFLD	56 (28.7%)
HCV	4 (2.0%)
Others	52 (26.6%)
Alcohol Consumption	
None	122 (62.6%)
Mild drinking	36 (18.5%)
Moderate drinking	37 (18.9%)
Diabetes	37 (18.9%)
Hypertension	62 (31.8%)
Histologic fibrosis stage	
F0	44 (22.6%)
F1	58 (29.7%)
F2	33 (16.9%)
F3	25 (12.8%)
F4	35 (17.9%)
Histologic inflammation grade	
G0	58 (29.7%)
G1	83 (42.5%)
G2	47 (24.1%)
G3	7 (3.5%)
SWE S8 (kPa)	7.22 ± 2.89
SWE E9 (kPa)	7.28 ± 2.70
Transient elastography (kPa)	10.39 ± 9.98
Laboratory test	
AST (U/L)	59 ± 40
ALT (U/L)	70 ± 57
AST/ALT ratio	1.37 ± 1.37
Platelet (10^3^/mm)	207 ± 76
Total bilirubin (mg/dL)	1.21 ± 1.89
Serum albumin (mg/dL)	3.91 ± 0.51
Serum creatinine (mg/dL)	0.88 ± 0.27
Prothrombin time (INR)	1.05 ± 0.12

**Table 2:** Correlation of histopathologic stage between S8 2D-SWE, E9 2D-SWE and TE.

	Histopathologic Stage	2D-SWE (S8)	2D-SWE (E9)	TE
Histopathologic stage		0.841 *	0.726 *	0.623 *
2D-SWE (S8)	0.841 *		0.819 *	0.840 *
2D-SWE (E9)	0.726 *	0.819 *		0.726 *
TE	0.623 *	0.840 *	0.726 *	

Abbreviations: *n*, number; SWE, shear wave elastography; TE, transient elastography; * *p* < 0.001.

**Table 3:** Recommended cut-off value of S8, E9 2D-SWE and TE for predicting liver fibrosis stage.

Fibrosis Stage	≥F1 (95% CI)	≥F2 (95% CI)	≥F3 (95% CI)	F = 4 (95% CI)
2D-SWE (S8)				
Cut-off, kPa	5.980	6.695	7.255	9.150
Sensitivity, %	71.52	79.57	86.67	82.86
Specificity, %	93.18	87.25	83.70	95.00
PPV, %	97.30	85.06	70.27	78.38
NPV, %	48.81	82.41	93.39	96.20
AUROC	0.887 (0.839–0.929)	0.908 (0.874–0.940)	0.905 (0.864–0.954)	0.931 (0.889–0.964)
2D-SWE (E9)				
Cut-off, kPa	5.720	6.420	7.355	8.875
Sensitivity, %	76.82	87.10	86.67	85.71
Specificity, %	86.36	81.37	80.74	0.918
PPV, %	95.08	81.00	66.67	69.77
NPV, %	52.05	87.37	93.16	96.71
AUROC	0.854 (0.797–0.903)	0.910 (0.871–0.944)	0.897 (0.844–0.939)	0.931 (0.874–0.969)
TE				
Cut-off, kPa	6.150	7.350	8.450	10.05
Sensitivity, %	77.48	84.95	85.00	91.43
Specificity, %	84.09	82.35	82.96	88.75
PPV, %	94.35	81.44	68.92	64.00
NPV, %	52.11	85.71	92.56	97.93
AUROC	0.867 (0.804–0.913)	0.887 (0.937–0.928)	0.907 (0.850–0.953)	0.951 (0.909–0.981)

Abbreviations: SWE, shear wave elastography; TE, transient elastography; PPV, positive predictive value; NPV, negative predictive value; AUROC, area under the receiver operating characteristic.

**Table 4:** Comparisons of classification by histologic stage between S8 2D-SWE, E9 2D-SWE and TE.

		Histologic Fibrosis Stage					
		0	1	2	3	4	Kappa
S8 fibrosis stage	0	41 (21.03)	32 (16.41)	6 (3.08)	4 (2.05)	1 (0.51)	0.635 (0.564–0.706)
	1	1 (0.51)	15 (7.69)	7 (3.59)	1 (0.51)		
	2	2 (1.03)	3 (1.54)	6 (3.08)	1 (0.51)	1 (0.51)	
	3		8 (4.10)	11 (5.64)	14 (7.18)	4 (2.05)	
	4			3 (1.54)	5 (2.56)	29 (14.87)	
E9 fibrosis stage	0	38 (19.5)	29 (14.9)	4 (2.04)	2 (1.03)		0.423 (0.381–0.465)
	1	2 (1.03)	14 (7.14)	3 (1.54)	2 (1.03)	1 (0.51)	
	2	2 (1.03)	7 (3.59)	10 (5.1)	2 (1.03)	1 (0.51)	
	3	2 (1.03)	7 (3.59)	10 (5.1)	13 (6.63)	3 (1.54)	
	4		1 (0.51)	6 (3.08)	6 (3.08)	30 (15.4)	
TE fibrosis stage	0	37 (18.9)	26 (13.33)	5 (2.56)	3 (1.53)		0.422 (0.337–0.511)
	1	4 (2.05)	17 (8.71)	4 (2.05)	1 (0.51)	1 (0.51)	
	2	2 (1.02)	6 (3.07)	11 (5.64)	3 (1.53)	1 (0.51)	
	3	1 (0.51)	7 (3.58)	8 (4.10)	8 (4.10)	1 (0.51)	
	4		2 (1.02)	5 (2.56)	10 (5.12)	32 (16.41)	

**Table 5:** Optimal cut-off values of liver stiffness measurement using SWE.

Aim	2D-SWE	Cut-Off	Sensitivity	Specificity	AUROC
TE < 10 kPa	2D-SWE (S8)	7.25	0.960	0.821	0.964
	2D-SWE (E9)	7.73	0.841	0.960	0.961
TE ≥ 15 kPa	2D-SWE (S8)	9.44	0.896	0.945	0.963
	2D-SWE (E9)	9.35	0.931	0.927	0.963

## Supplementary Materials

The following are available online at https://www.mdpi.com/article/10.3390/diagnostics11101817/s1.
