# Supplementary material for: The Diagnostic Accuracy of LOGIQ S8 and E9 Shear Wave Elastography for Staging Hepatic Fibrosis, in Comparison with Transient Elastography
Source: Diagnostics (Basel). 2021 Oct 1;11(10):1817. doi: 10.3390/diagnostics11101817 (PMC8535143; doi:10.3390/diagnostics11101817)
Supplement: Supplementary file 1 [file diagnostics-11-01817-s001.zip › diagnostics-1373125-supplementary.pdf]

Supplementary Table S1. In comparison with transient elastography (Fibroscan®).

	≥F1	≥F2	≥F3	F4
AUROC (95% CI) for TE	0.810(0.757-0.863)	0.834(0.782-0.887)	0.852(0.798-0.905)	0.889(0.824-0.955)
Comparison of ROC with S8 (p value)	0.449	0.273	0.927	0.318
Comparison of ROC with E9 (p value)	0.648	0.224	0.478	0.093

Abbreviations: AUROC, area under the receiver operating characteristic; TE, transient elastography.

Supplementary Table S2. Recommended cut-off value according to etiology.

Fibrosis Stage	≥F1 (95% CI)	≥F2 (95% CI)	≥F3 (95% CI)	F=4 (95% CI)
**Etiology: viral**				
**2D-SWE (S8)**				
Cut-off, *kPa*	5.880	6.890	7.440	8.850
Sensitivity, %	74.51	69.23	79.31	80.00
Specificity, %	100	92.86	91.67	92.11
PPV, %	100	96.43	92.00	80.00
NPV, %	13.33	52.00	78.57	92.11
AUROC	0.804 (0.676-0.941)	0.881 (0.779-0.957)	0.887 (0.781-0.954)	0.887 (0.766-0.974)
**2D-SWE (E9)**				
Cut-off, *kPa*	5.840	7.050	7.240	8.255
Sensitivity, %	76.47	71.79	82.76	93.33
Specificity, %	100	100	91.67	84.21
PPV, %	100	100	92.32	70.00
NPV, %	14.29	56.00	81.48	96.97
AUROC	0.784 (0.686-0.887)	0.873 (0.793-0.946)	0.885 (0.788-0.959)	0.919 (0.830-0.983)
**TE**				
Cut-off, *kPa*	6.150	7.200	7.200	10.00
Sensitivity, %	78.43	79.49	93.10	86.67
Specificity, %	100	85.71	75.00	89.47
PPV, %	100	93.94	81.82	76.47
NPV, %	15.38	60.00	90.00	94.44
AUROC	0.843 (0.730-0.941)	0.854 (0.758-0.927)	0.882 (0.770-0.970)	0.935 (0.860-0.993)
**Etiology: non-viral**				
**2D-SWE (S8)**				
Cut-off, *kPa*	5.985	6.970	7.835	9.330
Sensitivity, %	71.00	81.48	87.10	95.00
Specificity, %	92.86	89.77	88.29	95.90
PPV, %	95.95	83.02	67.50	77.27
NPV, %	57.35	88.76	96.08	97.50
AUROC	0.887 (0.827-0.939)	0.923 (0.876-0.970)	0.926 (0.869-0.973)	0.948 (0.915-0.979)
**2D-SWE (E9)**				
Cut-off, *kPa*	5.720	6.420	7.780	8.980
Sensitivity, %	75.00	94.44	87.10	90.00
Specificity, %	88.10	81.82	81.98	92.62
PPV, %	93.75	76.12	57.45	66.67
NPV, %	59.68	96.00	95.79	98.26
AUROC	0.847 (0.787-0.901)	0.931 (0.890-0.963)	0.924 (0.877-0.962)	0.940 (0.900-0.979)
**TE**				
Cut-off, *kPa*	6.000	7.350	9.800	12.150
Sensitivity, %	78.00	88.89	87.10	90.00
Specificity, %	83.33	81.82	93.69	95.08
PPV, %	91.76	75.00	79.41	75.00
NPV, %	61.40	92.31	96.30	98.31
AUROC	0.871 (0.811-0.934)	0.906 (0.848-0.956)	0.931 (0.874-0.980)	0.960 (0.915-0.994)

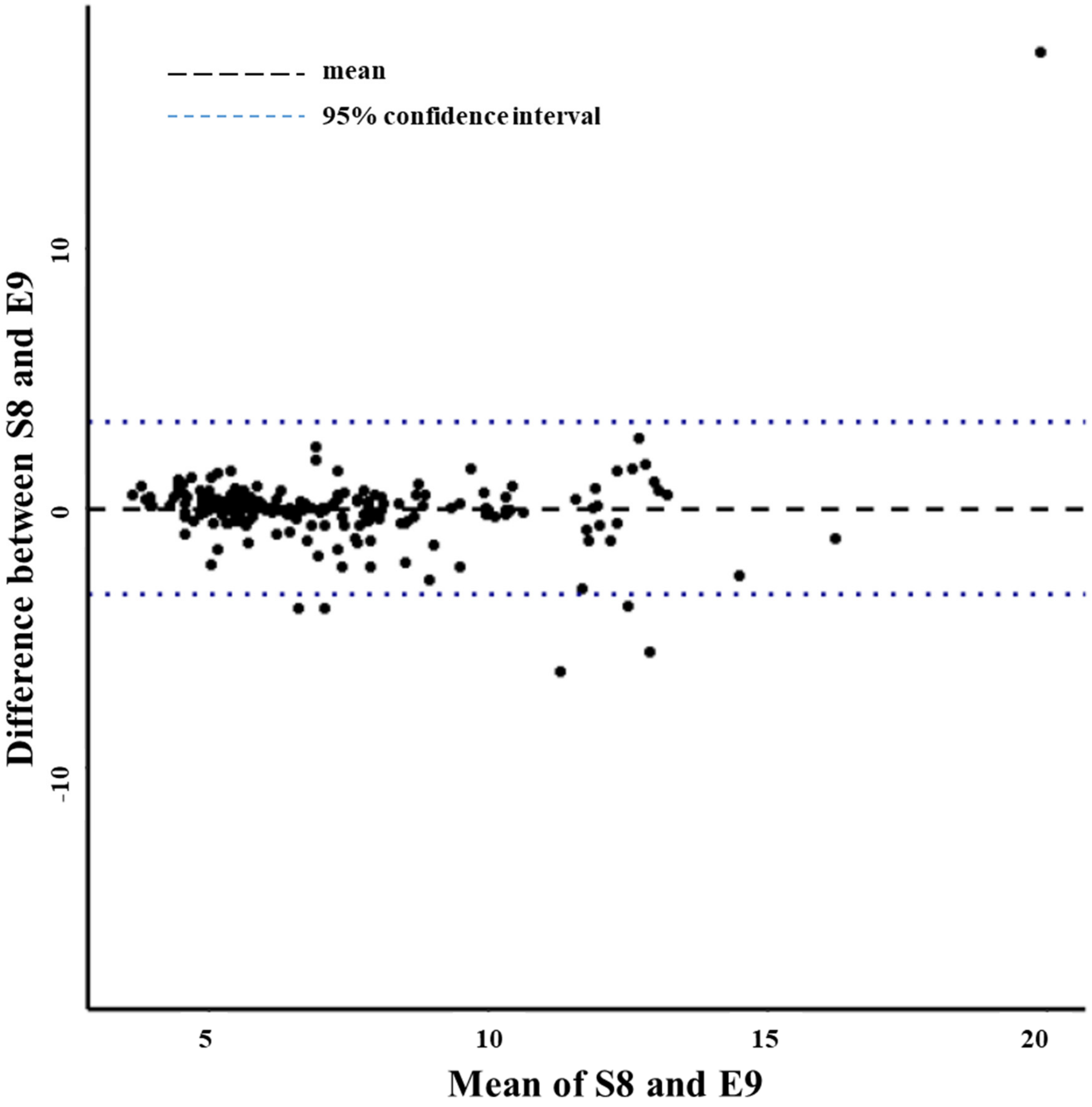

Supplementary Figure S1. Bland-Altmann plot.
